# Metabolic Engineering of Yeasts for the Production of the Triterpene Squalene: Current Status and Future Prospective

**DOI:** 10.3390/microorganisms13112422

**Published:** 2025-10-22

**Authors:** Shasha Zuo, Xinjia Tan, Jiwei Mao, Fanglin Hu, Jiaxu Chen, Liusha Fan, Qiyuan Lu, Yifei Zhao, Yongtong Wang, Zhiqiang Xiao, Siqi Zhang, Yang Shan, Juan Liu, Fuhua Fu

**Affiliations:** 1 Longping Branch, College of Biology, Hunan University, Changsha 410125, China; shashazuo@hnu.edu.cn (S.Z.); tanxinjia0209@163.com (X.T.); hfl20221225@163.com (F.H.); isfanls@163.com (L.F.); 18278952251@163.com (Q.L.); zhaoyifei@hnu.edu.cn (Y.Z.); quarknorthstar@163.com (Y.W.); zhangsiqi2021@hnu.edu.cn (S.Z.); sy6302@sohu.com (Y.S.); 2 Hunan Institute of Agricultural Product Processing and Quality Safety, Dongting Laboratory, Hunan Academy of Agricultural Sciences, Changsha 410125, China; chenjiaxu@hunaas.cn (J.C.); xiaozhiqiang@hnu.edu.cn (Z.X.); 3 Hunan Key Lab of Fruits & Vegetables Storage, Processing, Quality and Safety, Hunan Institute of Agricultural Product Processing and Quality Safety, Changsha 410125, China; 4 Department of Life Sciences, Chalmers University of Technology, SE41296 Gothenburg, Sweden; jiwei@chalmers.se; 5 College of Food Science and Nutritional Engineering, China Agricultural University, Beijing 100083, China

**Keywords:** squalene, microbial synthesis, metabolic engineering, *Saccharomyces cerevisiae*, *Yarrowia lipolytica*, application

## Abstract

Squalene, a linear triterpene compound characterized by its distinctive isoprenoid backbone with six transisoprene units, is widely used in the medicinal, nutraceutical, and cosmetic industries. The escalating global demand for squalene, coupled with growing ethical concerns over shark-derived sources and the inherent limitations of plant extraction (low yield) and chemical synthesis (environmental burden), has propelled microbial biosynthesis as a sustainable alternative. While substantial progress has been made in elucidating the mevalonate pathway and regulatory mechanisms of squalene biosynthesis, achieving industrially viable titers through microbial platforms remains an unresolved challenge. This review systematically summarizes recent advances in squalene biosynthesis using yeast chassis, with a focus on metabolic engineering strategies implemented in *Saccharomyces cerevisiae* and *Yarrowia lipolytica*. Furthermore, we elaborated on how squalene yields a diverse array of downstream derivatives through intricate enzymatic reactions. These derivatives—including triterpenoid saponins, triterpenoid acids, and steroids—exhibit significant applications in the pharmaceutical, nutraceutical, and cosmetic sectors. By integrating systems metabolic engineering with emerging synthetic biology tools, this work provides a roadmap for advancing strain engineering toward economically feasible squalene biomanufacturing.

## 1. Introduction

**Figure 1:**
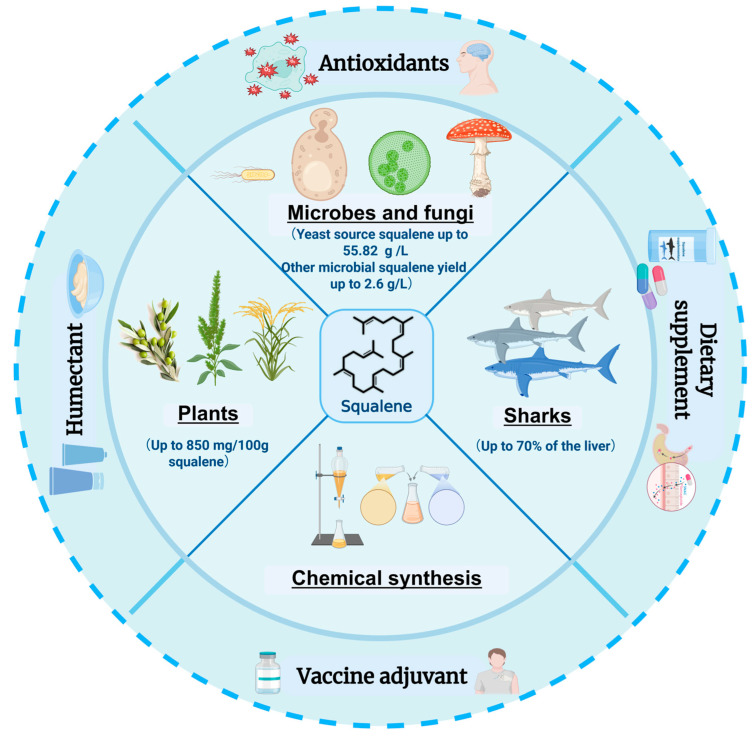
Applications and sources of squalene [4,15,16,17,18,19,20,21,22,23,24,25]. This figure was created using Biorender.com. The inner circle of the figure depicts the possible sources of squalene and its production, ranging from unicellular microorganisms such as yeast and bacteria, to multicellular fungi, plants, deep-sea sharks, and synthetic methods. The outer ring describes the main applications of squalene, including as antioxidants, moisturizers, vaccine adjuvants and dietary supplements.

Squalene, also known as triacontahexaene (C_30_H_50_), is a polyunsaturated hydrocarbon characterized by six isoprene double bonds, classifying it as a triterpene. It was first isolated from shark liver oil in 1916, and its name originates from the Latin word squalus, meaning shark [1]. This compound is naturally present in plants, fungi, insects, animals, and humans, among other organisms. Owing to its diverse biological activities—such as moisturizing, antioxidant, anti-aging, anti-fatigue, anti-inflammatory, and anti-tumor effects—squalene exhibits broad applicability [2,3,4,5]. It also functions as an immune enhancer, potentiating the immune response to specific antigens, and is employed as an adjuvant in vaccines, including those against influenza [6,7,8,9]. Given these versatile properties, the demand for squalene is increasing across various sectors such as cosmetics, food, medicinal health supplements, and personal care products (Figure 1) [4,7,10,11,12,13]. According to estimates by Mordor Intelligence, the global market value of squalene is projected to reach $167.15 million by 2024, with a compound annual growth rate of 5.96%, potentially climbing to $223.28 million by 2029 [14]. Thus, squalene demonstrates substantial potential in terms of both applications and market value.

In humans, squalene accounts for approximately 13% of sebum [26,27]. While it comprises less than 0.5% of the epidermis, it makes up 10% of surface lipids—a characteristic that underscores its efficient role in the lipid layer of the skin surface [28]. Squalene is primarily synthesized in the liver and secreted via the sebaceous glands [29,30]. The daily secretion of squalene varies between individuals due to dietary and genetic factors, with levels ranging from 125 to 475 mg per day [31]. Clinical studies have demonstrated that 60% to 85% of orally ingested squalene is effectively absorbed and subsequently distributed to various tissues [32]. For the skin in particular, it shields the skin against oxidative stress and free radicals [33]. However, squalene levels in the body start to decline after the age of 30, rendering supplementation—typically at a dose of 500 mg per day—advantageous for maintaining overall health and well-being [30,34,35].

Generally, squalene has been extracted from shark liver oil, with 3000 sharks required to obtain one ton of squalene [7]. The growing demand for squalene is endangering one-third of the world’s shark species, raising concerns about resource depletion and sustainability. Plant-derived squalene has attracted attention as a possible alternative. However, the long cultivation periods of plants, their low squalene content, and complex separation processes imply high costs and the need for abundant arable land [36,37,38,39]. In 1970, Johnsons et al. attempted the synthesis of squalene starting with 2,5-dimethoxy tetrahydrofuran, which was hydrolyzed from bis-diacetone alcohol [18,40]. The reaction route consisted of up to seven steps, and required complex conditions. In 1980, Scott et al. synthesized squalene from carvone, but the excessive complexity and number of steps meant that these attempts had little industrial value [41]. In recent years, microbial synthesis has emerged as an environmentally friendly, efficient, and sustainable alternative [42]. Yeast, which serves as a chassis cell for many bio-products, offers multiple advantages for the synthesis of squalene: its metabolic pathway is similar to that of eukaryotes, and it has a complete endoplasmic reticulum and secretion system for the accumulation of fat-soluble compounds. In fact, oleaginous yeasts are naturally rich in lipids and, thus, ideal for squalene production. Moreover, model yeasts such as *S. cerevisiae* are relatively easy to edit genes due to their high natural homologous recombination ability. The gene editing of *Yarrowia lipolytica* is more difficult than that of *S. cerevisiae*. However, efficient editing can now be achieved through CRISPR and homologous recombination optimization, enabling metabolic engineering strategies to optimize the biosynthesis of squalene [43,44,45]. These strains (i.e., *Y. lipolytica* and *S. cerevisiae*) are also suitable for stable, large-scale production due to their rapid growth, high cultivation density, and tolerance to industrial fermentation conditions, such as osmotic pressure and pH fluctuations. Several strategies have been demonstrated to be effective in enhancing squalene production in yeast, encompassing promoter engineering, organelle engineering, cofactor engineering, augmentation of precursor supply, knockout of competitive pathways, mitigation of squalene consumption, improvement of strain tolerance, promotion of extracellular secretion, and application of computer-aided design [46,47,48,49]. Yeasts show flexible metabolism by being able to use different carbon sources, including renewable materials like glucose and glycerol, which lowers costs in bioprocessing [50,51]. In addition, they are resistant to bacterial contamination, requiring fewer cleaning steps, making the industrial fermentation process easier [52,53]. With the rise of artificial intelligence (AI) and machine learning (ML), it has added a new dimension to bioengineering. The design of microbial cell factories relies on a large number of experimental trial and error, and the metabolic network is complex, with many variables, and the efficiency of manual screening is low. Through the innovation of AI and ML, the paradigm shift from “trial and error experiment” to “rational design” has been realized. Through enhanced ML, enzyme classification, enzyme or substrate properties prediction, optimal microenvironment prediction, and discovery of new enzymes or enzyme combinations with enhanced catalytic activities (e.g., AlphaFold2, RoseTTAFold2, CLEAN, Prekcat, etc.) were performed to achieve target product yield improvement [54]. In addition, AI deep learning was used to achieve reasonable metabolic pathway prediction and design. By using KEGG, MetaCyc, BIGG, KBase and other databases, biological information from multiple sources, such as genetic, molecular, physical and chemical, is integrated to make them available for metabolic pathway prediction. Computer simulations have been performed to enhance heterologous terpenoid production in *S. cerevisiae* using self-regulated gene knockdown strategies [55].

This review provides a comprehensive overview of the current knowledge regarding the biosynthesis of squalene in engineered yeasts, with particular emphasis on recent advancements involving *S. cerevisiae* and *Y. lipolytica*. These developments lay a theoretical foundation for future research in squalene biosynthesis. Given its wide range of existing application, yeast offers a mature platform for sustainable large-scale production of squalene.

## 2. Sources of Squalene

### 2.1. Squalene from Animal and Plant Sources

Traditionally, shark liver has been the primary source of squalene, accounting for 40–70% of its organ mass [4]. The rising global demand, however, has led to an estimated annual mortality of 3 to 6 million deep-sea sharks, posing severe risks to marine ecosystems. In response, the Convention on International Trade in Endangered Species of Wild Fauna and Flora has imposed strict bans on animal-derived squalene extraction, stimulating research into plant-based and microbial alternatives.

As shown in Table 1, which summarizes the squalene content from different plant sources. Olive oil contains 250–850 mg of squalene per 100 g of biomass, making it one of the richest plant sources available [17]. Amaranth seeds contain approximately 7% oil, while the proportion of squalene in oil can reach 6% to 8%., and amaranth seeds produce up to 470 mg/100 g of squalene [16,20,24]. Cold-pressed pumpkin seed oil usually has a lot of squalene, with levels up to 747 mg/100 g [15,21]. Argan oil has high levels (313 mg/100 g), as do traditional medicinal plants like tea leaves (28.9–368.2 mg/100 g) [19,23]. The amount of squalene in rice bran oil was 320 mg/100 g [22]. Nevertheless, plant-based extraction faces considerable limitations. Cultivation cycles for both plants and animals typically require six months to a year, rendering such sources relatively inefficient for scalable production. Moreover, the extraction process itself is not only costly but also environmentally detrimental, involving multiple steps and generating hazardous waste [56].

### 2.2. Chemical Synthesis of Squalene

**Figure 2:**
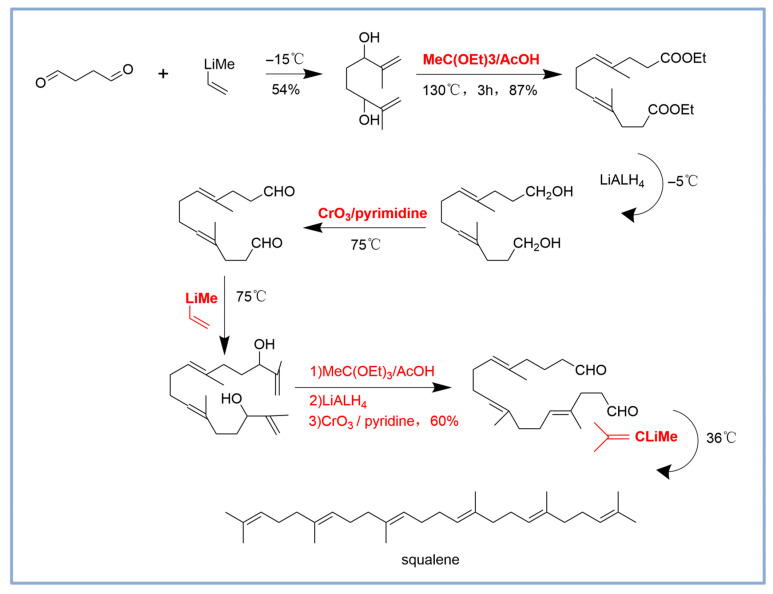
Chemical synthesis of squalene [18,40,41]. The parts marked in red and bold denote the key reagents and conditions employed in the reaction. They play specific roles at each step. As a strong nucleophile, 2-propenyllithium is utilized for the nucleophilic addition. MeC (OEt)_3_/AcOH is applied in esterification reactions to convert the enol into a diester compound. LiAlH is employed in the reduction of esters, converting the diester compound to a diol. CrO_3_/pyridine oxidizes alcohols to aldehydes or ketones.

The chemical synthesis of squalene encompasses multiple steps and reagents [18,40,41]. The starting material is succinaldehyde, this seven-step reaction involves multiple chemical transformation steps to progressively synthesize the target molecule through esterification, reduction, and oxidation reactions. In the first step, a nucleophilic addition reaction with the strong nucleophile 2-propenyllithium with succinaldehyde is used to attack one of the aldehyde groups in the succinaldehyde molecule, forming a new carbon-carbon bond and generating an intermediate. This was followed by esterification at 130 °C by using the MeC (OEt)_3_/AcOH; Aldehydes or ketones were then reduced to alcohols using LiAlH_4_ at −5 °C. Low temperature conditions were used in this step to ensure high selectivity of the reduction reaction and to reduce side reactions. Then, the alcohols were oxidized to aldehydes or ketones by the CrO_3_/pyridine, and the esterification, reduction and oxidation reactions were repeated. Finally, the molecular structure was fine adjusted through successive oxidation and reduction steps, and the target molecule was finally obtained. The whole process is synthesized through multiple functional group transformation and structural modification (Figure 2). The process is complicated. It has low yield and high cost. It also produces byproducts with similar structures. These factors make chemical synthesis of squalene more expensive. The product must undergo additional purification steps, which raises the cost even more. Hence, chemical synthesis remains at a disadvantage in terms of market competitiveness.

### 2.3. Squalene from Microbial Synthesis

In recent years, squalene has been successfully synthesized using *S. cerevisiae*, *Y. lipolytica*, and *Escherichia coli* [42,49,57,58,59,60,61,62,63,64,65,66]. While *E. coli* offers advantages, such as ease of genetic manipulation, a short growth cycle, and strong synthetic capacity, it has notable drawbacks. In particular, *E. coli* is unable to perform post-translational modifications on proteins after their synthesis—for instance, the attachment of sugar molecules (a process known as glycosylation). These post-translational modifications are essential for proteins from other species to fold into their correct conformation and exert proper biological functions. Additionally, the endotoxins produced by *E. coli* complicate the isolation and purification of squalene, making it less suitable for use in food and pharmaceuticals.

In contrast, the eukaryotic model organism *S. cerevisiae* presents several advantages for industrial production. These include robustness, safety, ease of genetic manipulation, low nutrient requirements, straightforward culture processes, and resistance to phage infections. Importantly, *S. cerevisiae* utilizes a process called the mevalonate (MVA) pathway. This pathway provides many building blocks, also called precursors, that are needed to make squalene. Examples of these precursors include isopentenyl diphosphate (IPP) and dimethylallyl pyrophosphate (DMAPP). *Y. lipolytica* may not be a traditional yeast, but it has a strong ability to break down fatty acids into acetyl-CoA, which is important in the production of certain substances. Additionally, *Y. lipolytica* is able to survive in salty water and extreme pH levels, as well as when it is in contact with organic compounds. The strong activity of its tricarboxylic acid cycle and pentose phosphate pathway supports the production of acetyl-CoA, ATP and NADPH, making it an excellent host for squalene synthesis.

## 3. Biosynthesis of Squalene in Yeast

The biosynthesis of squalene in yeast uses several enzymes. It starts with acetyl-CoA, a key building block in many cell processes. The first step combines two acetyl-CoA molecules, a reaction helped by the enzyme ERG10, to form acetoacetyl-CoA. This middle compound then reacts with another acetyl-CoA molecule, assisted by the enzyme ERG13, to create HMG-CoA. Finally, HMG-CoA is turned into MVA by the enzyme HMGR, using two NADPH energy molecules.

Next, the MVA pathway undergoes a series of phosphorylations and decarboxylations, resulting in the production of IPP. IPP is then isomerized to DMAPP, followed by the condensation of DMAPP and IPP to produce farnesyl pyrophosphate (FPP) in a reaction catalyzed by the enzyme ERG20. In the final step, the squalene synthase ERG9 located in the endoplasmic reticulum, catalyzes the conversion of FPP and NADPH into squalene.

**Figure 3:**
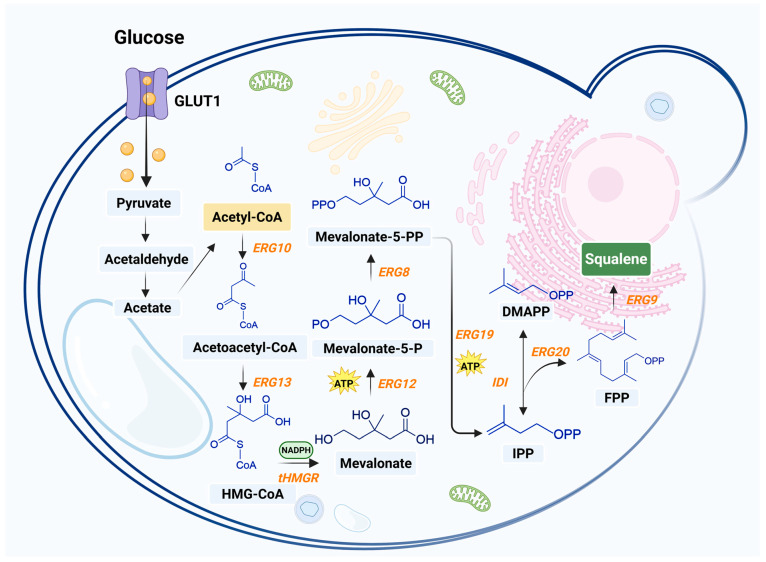
Biosynthesis of squalene in yeast [68,69,70,71]. This figure was created using Biorender.com. Black arrows denote endogenous pathways. Enzymes involved in squalene biosynthesis are listed in orange. The orange spheres represent glucose. The yellow box points to acetyl-CoA, a key precursor for squalene biosynthesis; the blue boxes represent various intermediates; and the green box denotes the target product (squalene). ERG10, acetyl-CoA C-acetyltransferase; ERG13, 3-hydroxy-3-methylglutaryl-CoA synthase; tHMGR, truncated 3-hydroxy-3-methylglutaryl-CoA reductase; ERG12, mevalonate kinase; ERG8, phosphomevalonate kinase; ERG19, diphosphomevalonate decarboxylase; IDI, isopentenyl-diphosphate delta-isomerase; ERG20, bifunctional (2E,6E)-farnesyl diphosphate synthase; ERG9, squalene synthase.

In the subsequent oxidation steps, squalene epoxidase ERG1 converts squalene into 2,3-oxidosqualene, which is eventually becomes ergosterol through a series of reactions (Figure 3). The biosynthesis of squalene in yeast begins in the cytoplasm, with the production of FPP via the MVA pathway; The process of converting FPP into squalene is primarily located within the endoplasmic reticulum. This spatial separation reflects the compartmentalization of metabolic pathways within yeast cells and the membrane-associated properties of the participating enzymes.

There are notable differences in enzyme structure, metabolic regulation, and flux between *S. cerevisiae* and *Y. lipolytica*. In the former, the MVA pathway is used solely for the synthesis of endogenous metabolites such as ergosterol [67]. In contrast, *Y. lipolytica* exhibits greater metabolic flexibility, enabling the MVA pathway to efficiently utilize various carbon sources and adapt to diverse growth conditions [56,64]. These differences highlight the distinct metabolic optimization strategies of the two yeasts, although the effectiveness of each pathway ultimately depends on the specific target product and the engineering strategies employed.

## 4. Metabolic Engineering Strategies for the Biosynthesis of Squalene in Yeast

**Figure 4:**
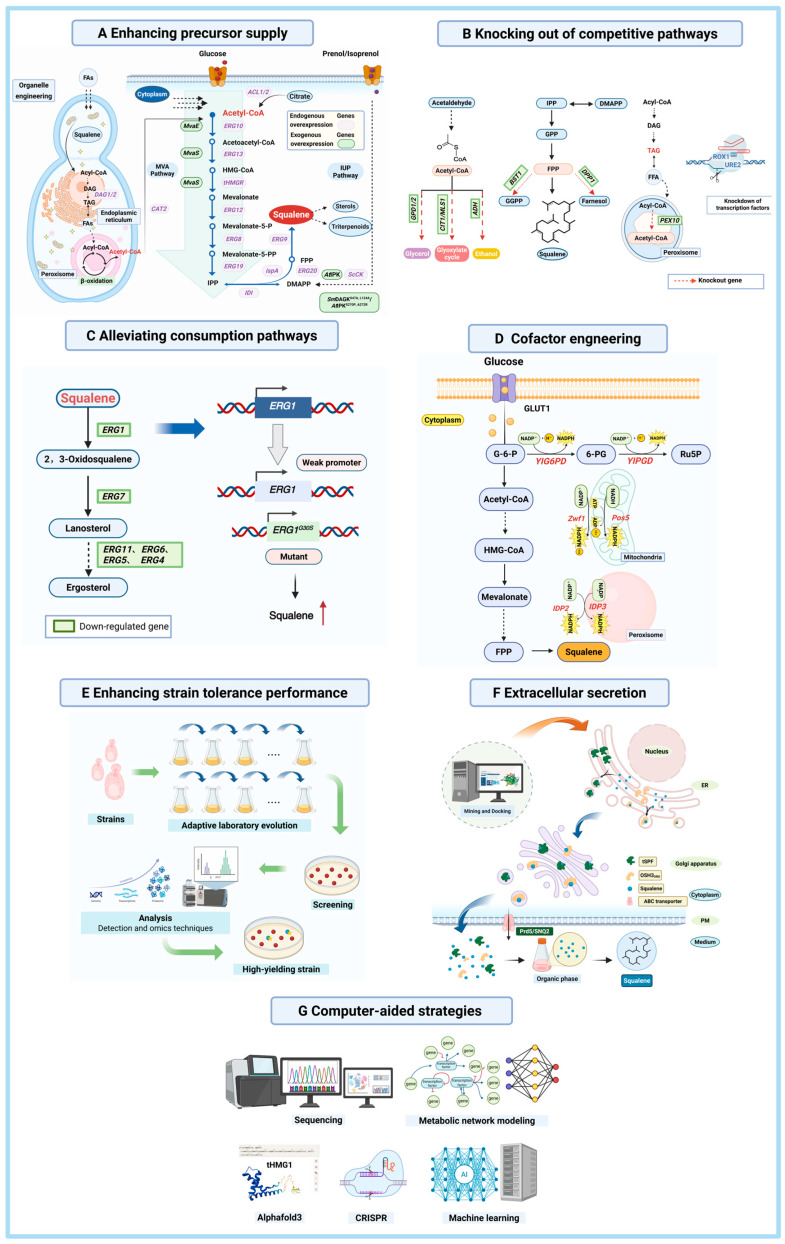
Metabolic engineering strategies for the biosynthesis of squalene in yeast [48,58,68,72,73,74,75,76,77]. This figure was created using Biorender.com. (**A**) Enhancing precursor supply. The glycolytic pathway generates acetyl-CoA, which enters the mevalonate (MVA) pathway. The supply of key precursors (acetyl-CoA, IPP, and DMAPP) is enhanced through endogenous gene overexpression, heterologous gene introduction, and organelle engineering. Glucose and isoprenol/prenol substrates are represented by red and purple spheres, respectively. (**B**) Knocking out competitive pathways. Byproduct formation is reduced by knocking out key genes in competing pathways (ethanol, glycerol, and glyoxylate cycle), thereby optimizing acetyl-CoA utilization. Squalene accumulation is further enhanced through regulation of transcription factors or knockdown of competing pathway genes. (**C**) Alleviating consumption. The consumption of squalene is limited by down-regulating or knocking down the related gene via weak promoters or mutations. Red arrow represents yield improvement (**D**) Cofactor engineering. The supply of cofactors, such as NADPH and ATP, can be ameliorated via overexpression of genes, such as *YIPGD* or *YIG6PD*, associated with cofactor production. (**E**) Enhancing strain tolerance. High-tolerance, high-yield strains are developed through adaptive laboratory evolution, involving random mutagenesis, domestication, high-throughput screening, and omics-assisted phenotypic optimization. (**F**) Extracellular secretion. Abbreviations: tSPF refers to the lipid-binding domain (amino acids 1–278) of transcription factor SPF, which participates in intracellular lipid transport and storage; OSH3_ORD_ denotes a fusion of OSH3 (an oxysterol-binding protein involved in lipid transport and ER membrane stability) and the ORD domain (which binds oxidized steroids and aids in cholesterol metabolism regulation); ABC transporters are transmembrane proteins that utilize ATP to transport substrates across membranes, with Pdr5 being a prominent plasma membrane ABC transporter in *Saccharomyces cerevisiae* known for drug efflux, and SNQ2 representing a potential endogenous ABC transporter in *Yarrowia lipolytica* involved in the efflux of lipids, metabolites, drugs, and toxic compounds. (**G**) Computer-aided strategies encompass computer simulation and AI tools to optimize metabolic engineering design. Tools include sequencing for genome and metabolic pathway profiling, Metabolic network modeling to help predict the optimal path, and ML (e.g., AlphaFold3) to predict protein structure or CRISPR-assisted gene editing.

In recent years, researchers have tried different ways to make squalene using yeast as a cell factory [47]. Common strategies include enhancing precursor supply, knocking out competitive pathways, alleviating consumption, cofactor engineering, enhancing strain tolerance, extracellular secretion and computer-aided design (Figure 4). As shown in Supplementary Material, which summarizes recent reports on the biosynthesis of squalene in yeast.

### 4.1. Enhancing Precursor Supply

Squalene and its precursors are predominantly synthesized via the MVA pathway. Consequently, enhancing the supply of these precursors represents a crucial strategy for increasing squalene yields [77]. A critical regulatory step in this pathway is the reduction of HMG-CoA to MVA, catalyzed by HMGR. In yeast, HMGR is encoded by two isozymes, HMG1 and HMG2, which share functional similarity but differ in subcellular localization and regulation. A widely adopted approach to enhance squalene production is the overexpression of *HMG1* or its truncated form, *tHMG1* [61,70,72,78,79,80]. Furthermore, combinatorial expression of *HMG1* with other enzymatic genes has been employed to augment precursor flux. For example, Kwak et al. overexpressed *tHMG1* and *ERG10* in conjunction with xylose feeding, achieving a squalene titer of 150 mg/L [81].

The transcription factor UPC2 helps to control the *ERG* genes that make ergosterol in yeast [82]. The UPC2-1 version has a small change (G888D) in its activation domain, and this change greatly boosts the production of terpenes [83,84,85]. The bacterial enzyme ispA, which is involved in isoprenoid biosynthesis, catalyzes the polymerization of isoprene units to IPP or FPP [76,86,87]. Co-expression of *ispA* with *tHMG1* in *S. cerevisiae* has resulted in 400 mg/L of squalene [86]. The metabolic capacity of *S. cerevisiae* for the MVA pathway can be expanded by catalyzing enzymes associated with lipid metabolism and stress response, such as the diacylglycerol acyltransferase (DGA1) and carnitine CIS acetyltransferase (CAT2). Wang et al. co-overexpressed *HMGR*, *ERG9*, and *ERG20*, followed by further co-overexpression of *DGA1* and *CAT2*, which increased squalene production in *Y. lipolytica* to 1381.4 mg/L [64]. Additionally, overexpression of *ACL1* and *ACL2*, which encode citrate ATP lyase from *Y. lipolytica*, significantly increased squalene production by boosting acetyl-CoA biosynthesis in *S. cerevisiae* [65].

In addition to modulating endogenous pathways, the introduction of exogenous pathways can effectively enhance precursor availability. Ma et al. introduced *MvaE* and *MvaS* from *Enterococcus faecalis* to enhance the metabolic flux through the upstream MVA pathway in *Y. lipolytica*, thereby increasing squalene content [69]. The same group also introduced an exogenous isopentenol utilization pathway into *S. cerevisiae*, which significantly shortened the biosynthesis route to IPP and DMAPP and established an efficient platform for terpenoid production [88]. Other researchers further improvements to this pathway came from molecular docking and enzyme-substrate structure analysis, which led to the co-overexpression of *SmDAGK^S47A^*^,L124A^ and *AtIPK^S270P,A272R^*, which increased the accumulation of squalene by 152.95% [89].

In a different approach, the human hypoxia-inducible factor complex was introduced into *S. cerevisiae*, triggering a Warburg effect similar to that observed in cancer cells. This complex enhances the accumulation of pyruvate in the cytoplasm, and because yeast lacks lactate dehydrogenase, the increased pyruvate promotes the pyruvate dehydrogenase bypass, ultimately boosting acetyl-CoA biosynthesis. This metabolic shift resulted in a 2.7-fold increase in squalene production compared to the control strain [90].

Organelle engineering has emerged as a key strategy to improve squalene biosynthesis by optimizing the structure and function of organelles, enhancing metabolic flow, and improving energy utilization. Kim et al. overexpressed *INO2*, a key regulator of endoplasmic reticulum size in *S. cerevisiae*, resulting in a larger reticulum and greater squalene production [91]. At the same time, Liu et al. increased the activity of several enzymes, including ERG10, ERG13, tHMG1, ERG12, ERG8, MVD1, IDI1, ERG20, ERG9, acetyl-CoA synthase (ACS1), and citrate lyase (ACL). They did this in peroxisomes to increase the production of acetyl-CoA [92]. Similarly, some researchers overexpressed these same genes in mitochondria [71]. Another method of used cytoplasm-peroxisome linkage engineering, which incorporates peroxisome signal peptide screening, was used to overexpress peroxisomal genes *POT1*, *PXA1/2*, and *POX1/2*. This approach helped optimize carbon flux and prevent metabolic drain from the MVA pathway [70]. Notably, Tang et al. implemented simultaneous engineering of multiple organelles, achieving a remarkable squalene concentration of 55.8 g/L in *S. cerevisiae*—the highest titer reported to date [25].

### 4.2. Knockout of Competitive Pathways

Disrupting competing metabolic pathways is a highly effective strategy to increase squalene production. Knockout of *ERG6* and *ERG11*, which are involved in ergosterol biosynthesis, has been shown to increase squalene production to 43 mg/g DCW [48,74]. Some researchers also combined knockout of downstream genes about ergosterol biosynthesis pathway along with that of *ADH*, which is involved in ethanol production, resulted in 304.49 mg/L squalene [93]. Wei et al. induced lipid accumulation and increased squalene accumulation by knocking out the peroxisomal membrane E3 ubiquitin ligase *PEX10* and *URE2*, a transcriptional regulator responsible for the inhibition of nitrogen catabolism in *Y. lipolytica* [65]. Additionally, acetyl-CoA consumption has been reduced through knockout of *CIT2* and *MLS1* [64]. Furthermore, deletion of the *ROX1* gene, which encodes a transcriptional repressor of ergosterol biosynthesis, has been shown to enhance both the MVA pathway and terpenoid synthesis in *S. cerevisiae* by derepressing the overall metabolic flux [94,95].

### 4.3. Alleviating Consumption Pathways

Reducing metabolic flux through competing consumption pathways is a crucial strategy for improving the yield and efficiency of target metabolites. By optimizing these pathways, metabolic flux could be redirected toward the desired product, thus enhancing overall biosynthetic efficiency.

An effective strategy involves converting ethanol to acetyl-CoA. Li et al. demonstrated that overexpressing *ADH2* (alcohol dehydrogenase) in *S. cerevisiae*, along with heterologous expression of the transcriptional activator *ADA* from *Dickeya zeae*, promoted the conversion of acetaldehyde to acetyl-CoA [58]. ADA does not require ATP, alleviating the metabolic burden on the cell and enhancing the efficiency of squalene biosynthesis.

In the native metabolic pathways of *S. cerevisiae*, squalene is formed mainly as an intermediate in ergosterol biosynthesis. It is quickly converted into 2,3-oxidosqualene by *ERG1* and then further metabolized to produce ergosterol. In unmodified wild-type strains, squalene levels are typically undetectable due to its swift diversion to ergosterol biosynthesis. Because ergosterol is a necessary component of the yeast cell membrane and is essential for cell growth and reproduction, simply knocking out genes involved in ergosterol synthesis is not a viable option [96]. To overcome this issue, promoter engineering can be employed to regulate *ERG1* expression. Kamimura et al. achieved a squalene yield of 5 mg/g DCW by introducing random mutations into *ERG1*, effectively reducing the flux through the ergosterol biosynthesis pathway [97]. Zhou et al. further engineered *pERG1* in *S. cerevisiae* by replacing the SRE sequence with the marO sequence and inserting marO after the TATA box. This modification reduced *ERG1* expression and promoted squalene accumulation. Additionally, they introduced the *ERG1^G30S^* mutant to further minimize squalene consumption [98]. Lu et al. replaced the endogenous ERG1 promoter with a homolog from *Phaffa rhodozyma* to down-regulate *ERG1*, whereas Manzoor et al. used a weaker *pERG1* [61,99]. *ERG1* expression can be modulated also by transcription factors. Jorda et al. found that knocking out the *ROX1* transcriptional repressor or the post-transcriptional repressors *CTH1* and *CTH2*, increased *ERG1* expression. Conversely, downregulating these repressors had the opposite effect [100].

In summary, a combination of genetic modifications and metabolic engineering strategies, such as promoter engineering, gene knockout, and transcription factor modulation, can effectively alleviate consumption pathways and redirect metabolic flux toward the production of valuable metabolites such as squalene.

### 4.4. Cofactor Engineering

Cofactor engineering is essential for optimizing biosynthetic processes, particularly in improving the efficiency and yield of target metabolites. Cofactors such as NAD (P)H and ATP mediate the activity and stability of metabolic pathways [73]. Introduction or overexpression of key genes such as NADH-HMG-CoA reductase (NADH-HMGR) to alleviate cofactor stress in the metabolic pathway boosts the concentration of target metabolites including squalene [101,102]. For instance, Li et al. tackled the limited availability of NADPH in the MVA pathway by introducing NADH-HMGR from *Silicibacter pomeroyi.* This alleviated cofactor pressure and resulted in 11.05% higher squalene production [58]. Paramasivan et al. enhanced *Pos5* and *Zwf1* expression in *S. cerevisiae* [103]. And then co-expressed them with other genes to boost the NADPH supply, eventually achieving 27.5-fold more squalene compared to control strains [79]. Similarly, Liu et al. overexpressed *G6PD* and *PGD*—two key pentose phosphate pathway genes—in *Y. lipolytica*, significantly elevating squalene synthesis [78]. Additionally, Liu et al. improved squalene titers by optimizing the supply of both NADPH and acetyl-CoA [60]. Beyond central carbon metabolism, the expression of specific isocitrate dehydrogenases such as IDP2 and IDP3 can also support NADPH generation required for mitochondrial β-oxidation of fatty acids [92,104].

### 4.5. Enhancing Strain Tolerance

Enhancing strain tolerance represents a pivotal metabolic engineering strategy, enabling sustainable synthesis of target molecules and facilitating the rational design of robust microbial cell factories [76]. In *S. cerevisiae*, a key approach to boosting squalene production is by reducing the squalene epoxidase activity. This can be achieved using non-competitive inhibitors such as terbinafine, an antifungal drug whose structure resembles squalene. Terbinafine competes with squalene for binding to the enzyme ERG1, thereby inhibiting the formation of 2,3-oxidosqualene, a precursor in the squalene biosynthetic pathway [105,106]. This specific inhibition mechanism enables terbinafine to serve as a selective pressure during adaptive laboratory evolution, enriching for engineered *S. cerevisiae* exhibiting enhanced squalene flux and improved accumulation [42]. Such approaches are crucial for advancing the adaptive evolution of *S. cerevisiae* strains designed for efficient squalene production, as well as for cultivating more robust, high-yield strains. In a complementary approach, using another approach, Son et al. adapted cells to elevated squalene levels by reinforcing the yeast cell wall and minimizing the dose-dependent inhibitory effect of squalene on growth [107]. While the use of ethanol as a carbon source during fermentation has been shown to increase squalene accumulation, high ethanol concentrations can negatively affect both strain growth and product accumulation. To address this conundrum, researchers have implemented adaptive evolution under progressively increasing ethanol stress, successfully obtaining strains with enhanced ethanol tolerance and higher squalene yields [108].

### 4.6. Extracellular Secretion

Squalene is an extremely hydrophobic compound. Given its pronounced hydrophobicity and considerable molecular size, the lipid bilayer of the cell membrane effectively restricts its passive diffusion. In yeast, efficient transport of squalene across the cell membrane is challenging, preventing natural extracellular secretion. Additionally, intracellular accumulation of squalene can disrupt the fluidity and stability of the cell membrane, further complicating its extracellular transport. Excessive buildup of squalene inside cells may also have toxic effects, damaging cellular structure and function.

To overcome these limitations, specialized transport systems or binding proteins are typically required to facilitate squalene’s passage through the cell membrane. Utilizing appropriate signal peptides or binding proteins can enhance the interaction between squalene and transport proteins, improving both binding and transport efficiency. This promotes efficient secretion of squalene from the intracellular to the extracellular environment, preventing excessive intracellular accumulation and reducing metabolic stress on the cells. Ultimately, this approach aims to increase overall squalene production. Son et al. enhanced squalene production by systematically integrating Suc2-tSPF into the original production strain, achieving a yield of 69 mg/g DCW—nearly three orders of magnitude higher than the wild-type strain [109]. Liu et al. identified the ATP-binding cassette transporter Pdr5 and the oxysterol-binding protein Osh3 as key facilitators of squalene efflux. Through a comprehensive “mining-docking-construction-validation” process, they demonstrated that strains overexpressing Pdr5 and Osh3 showed a 141.1-fold greater squalene production compared to control strains [110]. Chai et al. screened that the most significant secretion of squalene was observed when SNQ2 was bound to OSH3. Additionally, they developed a carrier protein-mediated metabolite transport system by fusing the OSH3 binding region to a secreted signal peptide, further enhancing squalene efflux [68].

### 4.7. Computer-Aided Strategies

Computer-aided strategies offer powerful methods for modeling metabolic networks in yeast, identifying bottlenecks, and targeting modifications to enhance squalene production [75]. Zhang et al. designed a gene silencing strategy based on autonomous oscillation, integrating computational and synthetic biology. By combining genome-scale metabolic models of *S. cerevisiae* with advanced algorithms such as flux balance analysis and OptKnock, they optimized the production of pentacyclic triterpenoids [55]. Liu et al. utilized databases such TransportDB2 to identify potential squalene transporters in *S. cerevisiae* [110]. Systems- and model-based metabolic engineering can enhance product formation by predicting gene knockdown and overexpression targets. Using the novel metaheuristic tool FOCuS to predict some key gene knockout targets, including *LYS1*, *GAP1*, *AAT1*, and *PDC1*, it was possible to increase squalene synthesis by 2.23-fold and 4.24-fold, respectively, compared with the control. These findings offer important strategies for overcoming squalene production bottlenecks, which is a critical challenge in microbial cell factory design and natural product biosynthesis [75].

## 5. Applications

**Figure 5:**
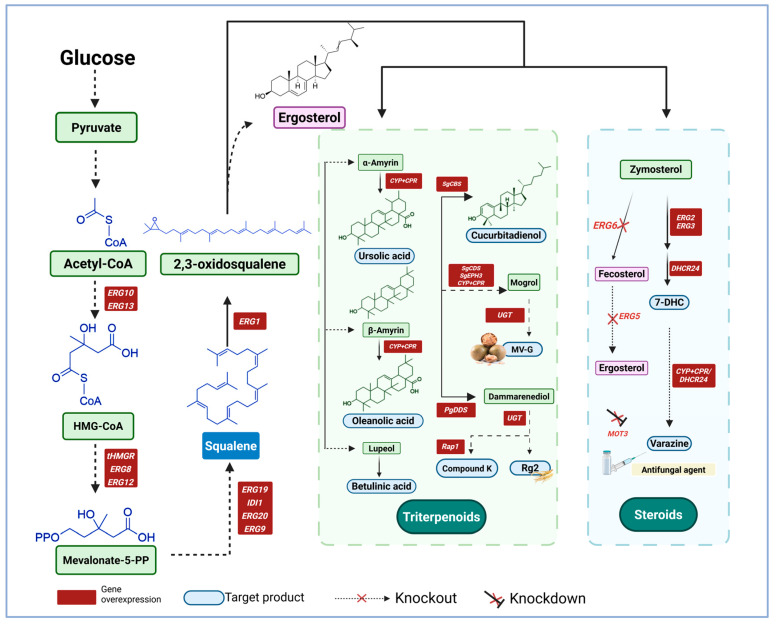
Downstream applications of squalene [111,112,113,115,118,119,120,125,126,127,128,129,130,131,132,133,134,135,136,137,138,139,140,141,142]. This section mainly mentions the application of squalene in the biosynthesis of Terpenoids and steroids, and the main enzymes involved in the reaction process.

As a natural triterpenoid, squalene is the precursor of many important bioactive compounds. Its unique chemical structure generates a variety of downstream products through complex enzymatic reactions in vivo (Table 2). Triterpenoids and steroids are important derivatives of squalene, with applications in medicine, healthcare, and cosmetics (Figure 5).

### 5.1. Triterpenoids

Squalene is a precursor for the synthesis of triterpenoids, such as ursolic acid, oleanolic acid, ginsenoside and mogroside V (MG-V), etc. Ursolic and oleanolic acids possess anti-cancer, antioxidant, and so on [143,144]. The synthesis of them starts with squalene, and is followed by a series of oxidation and carboxylation reactions. These biologically active compounds have significant clinical value as therapeutics against inflammation, tumors, liver diseases, and hypoglycemia, and are widely used in botanicals and nutraceuticals. In synthetic biology, it is common to overexpress key enzyme genes, optimize the expression efficiency of heterologous genes, and knock out genes of competitive metabolic pathways to enhance the flux of target metabolites [135,138]. These steps are coupled with the optimization of fermentation processes (e.g., adjusting the initial sugar concentration and implementing fed-batch fermentation) to further augment the yield of target products. Lu et al. introduced amyrin C-28 oxidase and cytochrome P450 reductase to optimize the titers and ratios of ursolic and oleanolic acids. Through a glucose and ethanol fed-batch fermentation strategy, the final titers of the two acids reached 123.27 and 155.58 mg/L, respectively, with yields that were 4.77 and 4.95-fold higher than in the original strain [111]. Jin et al. ultimately achieved titers of 692.3 mg/L and 253.4 mg/L for ursolic and oleanolic acids in shake flasks, and 1132.9 mg/L and 433.9 mg/L in 3 L bioreactor through lipid droplet compartmentalization of CrAO and AtCPR1, which improved NADPH regeneration [112]. Jia et al. obtained 2.33 g/L ursolic acid in a 5 L bioreactor by screening efficient CYP450 and compatible CPR, as well as by optimizing cofactors and metabolic pathways. The resulting yield was 70-fold higher than in the original strain [113]. Recently, by constructing a heterologous synthesis module, optimizing the mevalonate, carbon flow balance and so on, the yield of the engineered strain reached 1083.62 mg/L in shake flask culture, and increased to 8.59 g/L in a 5 L bioreactor. This is the highest microbial synthesis ever recorded [114]. Moreover, the yield of oleanolic acid has been successfully increased by a range of different methods. Some sought to optimize electron transfer efficiency through fusion protein expression, achieving a fed-batch fermentation yield of 540.7 mg/L oleanolic acid in a 5 L bioreactor [115]. Moreover, a 7.6-fold increase in oleanolic acid production was attained by reconstructing metabolic pathways or the galactose regulatory network, thereby enhancing the expression of heterologous genes to dynamically balance cellular metabolism [138]. Another study employed computational biology to design a gene autonomous oscillatory silencing strategy based on global metabolic flux simulation and applied it in *S. cerevisiae* to promote stable oleanolic acid production [55]. A comprehensive comparison of different *β*-amyrin synthases and CYP716A subfamilies, provides a reference for future engineering of both *S. cerevisiae* and *Y. lipolytica* [136]. In addition, there have been some studies on betulinic acid (BA) biosynthesis. BA is a lupane-type triterpenoid that exhibits remarkable anti-cancer and anti-HIV properties. In the innovative study of *S. cerevisiae*, the researchers cleverly established a second synthesis pathway in the peroxisome, successfully realized the synergistic regulation of the two pathways, by further optimizing the fed-batch fermentation process, the yield of BA was finally increased to 682.29 mg/L, which was 48.7-fold higher than the initial level [116]. What is more, the dual engineering strategy of combining peroxisomes and lipid droplets has been studied to optimize the synthesis and storage of hydrophobic BA. The enzymes BPLO and ATR1 were used to construct BA synthesis pathway. By optimizing the expression of *BPLO* and *ATR1* linker peptide, BA production reached 205.74 mg/L in fed-batch fermentation in 5 L bioreactor [117].

Rare-types ginsenosides are triterpenoid saponins extracted from ginseng. Unlike common ginsenosides, rare ginsenosides have unique glycosylation structures, usually containing some special sugar groups or linkages to these groups, which confer specific biological activity. Rare ginsenosides have anti-fatigue, anti-aging, anti-cancer, and immunoregulatory properties, which could be exploited in clinical practice for the treatment of cancer, cardiovascular disease, and aging [145,146,147,148]. Metabolic engineering, enzyme modification, and fermentation process optimization have significantly improved the production of Rh2 and other rare ginsenosides [139,140]. Specific engineering steps involve increase the supply of the precursor protopanaxiadol and improving glycosylation efficiency by optimizing the MVA pathway and the expression of key UDP-glycosyltransferases (UGTs). A maximum Rh2 yield of 2.25 g/L in a 10 L fermenter has been achieved through modular optimization and UGTPg45 modification [118]. Similarly, boosting the catalytic efficiency of UGT51 by approximately 1800-fold through semi-rational design, in combination with glycosylation and precursor optimization, has led to 300 mg/L Rh2 in a 5 L bioreactor [119]. Zhang et al. constructed a heterologous xylose metabolic pathway, using xylose and mixed sugar medium, with which they achieved 1.47 g/L Rh2 in a fermenter [120]. Their results opened the way for the utilization of renewable carbon sources in steroid biosynthesis. A yield of 354.69 mg/L Rh2 and a glycosylation ratio of 60.4% were reported in culture flasks following screening for efficient UGTs and heterologous expression [141]. In addition, researchers increased the production of Dammarenediol-II by 37.5-fold by enhancing the expression of *ERG1* gene to increase the supply of 2,3-oxidosqualene, down-regulated the expression of *ERG7* gene to reduce competitive consumption, and exogenously supplemented squalene, which provided an optimized platform for the efficient biosynthesis of ginsenosides [121]. Moreover, overexpression of Rap1 increased the production of Compound K, a downstream product of Dammarenediol-II, by 4.5-fold [122]. What is more, MG-V, as a cucurbitane triterpene saponins, is 400-fold sweeter than sucrose, and has anti-inflammatory, neuroprotective and other medicinal values. De novo biosynthesis of triterpene saponins MG-V was achieved by the addition of two glycosyltransferases to a modified *S. cerevisiae* strain. The yield reached 10.25 mg/L in shake flask and 28.62 mg/L in a 5 L bioreactor [123]. The accumulation of cucurbitadienol as a precursor for MG-V was increased to 6.19 /L in a 5 L bioreactor through a multi-module strategy, which is the highest titer reported so far [124]. These studies have demonstrated innovative strategies for the industrial production of ginsenosides and other rare triterpenoid saponins through systematic optimization.

### 5.2. Steroids

7-Dehydrocholesterol (7-DHC) is a sterol found in animals and precursor for synthesizing cholesterol and also the ecdysone hormone, it can be directly converted into vitamin D3 after exposure to ultraviolet light [128]. 7-DHC is widely used in the food and pharmaceutical industries [132]. We can increase how much 7-DHC we get by using genetic engineering and metabolic optimization. For example, by optimizing the inducible GAL promoter, integrating multiple copies of key enzyme genes and knockout NEM1, the yield of 7-DHC reached 1.07 g/L [125]. Another study achieved 360.6 mg/L 7-DHC yield in shake flask by compartmentalization and modular metabolic remodeling [126]. Moreover, blocking the competing pathways by knocking out the *ERG5* and *ERG6* genes and integrating two copies of the *DHCR24* gene from *Gallus cucurbitacin*, along with knockout of *MOT3* and the overexpression of key enzymes, elevated the yield of 7-DHC to 2.0 g/L [128]. A modular metabolic remodeling strategy, whereby the metabolic pathway was divided into central metabolism, MVA pathway, squalene pathway, and 7-DHC synthesis module, coupled with endoplasmic reticulum compartmentalization, successfully raised the 7-DHC production in a fermenter to 2.87 g/L [129]. In another study, a 7-DHC yield of 1.328 g/L was achieved by dynamically suppressing the *ERG6* gene and using the Ty1 transposon to increase the copy numbers of *ERG1* and *DHCR24* [127]. Furthermore, Bi et al. achieved 640.77 mg/L in shake flask and 4.28 g/L through strategies such as peroxidase body localization and enhancement of NADPH [131]. Some researchers also rebalancing the redox state and integrating multiple copies of key enzyme genes increased the 7-DHC yield in a shake flask to 867.6 mg/L [130]. Finally, Xiu et al. directed the squalene posterior pathway towards lipid droplets and further alleviated the alleviating redox imbalance, increasing the yield of 7-DHC to 5.1 g/L, which is the highest yield reported to date [132]. Taken together, these approaches increase the prospects for the efficient microbial production of 7-DHC at industrial scale.

Additional promise comes from the development of production platforms for 7-DHC derivatives. For instance, blocking competing pathways, overexpressing key MVA pathway genes, manipulating lipid metabolism, and enhancing the supply of the cofactor 3-phosphoadenosine-5 phosphosulfate, raised the cholesterol sulfate yield to 545 mg/L in fed-batch fermenters [133]. Interesting, 7-DHC was used also as a precursor to reconstruct the five-step verazine biosynthesis pathway. The procedure required heterologous expression of eight enzymes from seven different species, and yielded 83 μg/L verazine, a potent antifungal agent [134].

## 6. Summary and Outlook

Shark liver oil restrictions and the limited yields from plant sources have accelerated the shift toward microbial production of squalene. However, several challenges remain, including low titers, cellular toxicity, and difficulties in scaling up processes. This review systematically summarizes recent advances in yeast-based squalene biosynthesis, focusing on seven key engineering strategies: enhancing precursor supply, blocking competitive pathways, cofactor engineering, reducing metabolic consumption, improving strain tolerance, promoting extracellular secretion, and computer-aided design. We also highlight applications in sustainable manufacturing and associated environmental benefits. Machine learning integration with metabolic engineering and omics data aids strain optimization, but high-throughput method gaps limit efficiency. Standardizing biological datasets via knowledge engineering can train models to predict gene–enzyme dose effects and identify robust cell factories. Coupling ML with responsive biosensors may revolutionize squalene and triterpenoid biosynthesis, advancing sustainable microbial production. In recent years, AI-driven biomanufacturing is a hot spot, and some progress has been made. For example, Bo Wang’s team from the University of Toronto recently proposed a model MFM that can be predicted and trained in genomics, transcriptomics, etc., and can be applied to a variety of downstream tasks through transfer learning. The continuous development of these technologies lays a solid foundation for future intelligent production, making the realization of a fully self-driven laboratory just around the corner! Nevertheless, the integration of AI into biological design faces several hurdles, including the need for large, high-quality datasets and interdisciplinary expertise. Technical barriers, regulatory frameworks, and data privacy issues also present challenges that require collaborative efforts across academia and industry to overcome. In summary, AI and ML will push biomaterial from “experience driven” to “data driven” and reshape the industrial ecology through agents, multimodal models, and self-evolving systems!

## Figures and Tables

**Table 1:** Squalene content from different plant sources.

Plant Origin	Squalene Content (mg/100 g)	References
**Olive oil**	250–850	[17]
**Amaranth seeds**	Up to 470	[16,20,24]
**Pumpkin seeds oil**	Up to 747	[15,21]
**Argan oil**	Up to 313	[19]
**Tea leaves**	28.9–368.2	[23]
**Rice bran oil**	Up to 320	[22]

**Table 2:** The downstream applications and production of squalene.

Product	Yield		References
	Shaking Flask	Fermentation	
Ursolic acid	/	123.27 mg/L	[111]
	692.3 mg/L	1132.9 mg/L	[112]
	/	2.33 g/L	[113]
	1083.62 mg/L	8.59 g/L	[114]
Oleanolic acid	/	155.58 mg/L	[111]
	253.4 mg/L	433.9 mg/L	[112]
	/	540.7 mg/L	[115]
Betulinic acid	/	682.29 mg/L	[116]
	/	205.74 mg/L	[117]
Rh2	/	2.25 g/L	[118]
	/	300 mg/L	[119]
	/	1.47 g/L	[120]
Dammarenediol-II	/	37.5-fold	[121]
Compound K	/	4.5-fold	[122]
MG-V	10.25 mg/L	28.62 mg/L	[123]
Cucurbitadienol	/	6.19 g /L	[124]
7-DHC	/	1.07 g/L	[125]
	360.6 mg/L		[126]
	/	1.328 g/L	[127]
	/	2.0 g/L	[128]
	/	2.87 g/L	[129]
	/	867.6 mg/L	[130]
	640.77 mg/L	4.28 g/L	[131]
	/	5.1 g/L	[132]
Cholesterol sulfate	/	545 mg/L	[133]
Verazine	83 μg/L		[134]

## Data Availability

No new data were created or analyzed in this study. Data sharing is not applicable to this article.

## Supplementary Materials

The following supporting information can be downloaded at: https://www.mdpi.com/article/10.3390/microorganisms13112422/s1, Table S1. Research progress in the biosynthesis of squalene in yeast. References [25,68,69,70,71,92,110,149,150,151] are cited in the Supplementary Materials.
