# Supplementary material for: Metabolic Engineering of Yeasts for the Production of the Triterpene Squalene: Current Status and Future Prospective
Source: Microorganisms. 2025 Oct 22;13(11):2422. doi: 10.3390/microorganisms13112422 (PMC12654689; doi:10.3390/microorganisms13112422)
Supplement: Supplementary file 1 [file microorganisms-13-02422-s001.zip › microorganisms-3914535-supplementary.pdf]

Table S1. Research progress in the biosynthesis of squalene in yeast.

Strain	Strategies	Yield		References
		Shaking flask	Fermentation	
S. cerevisiae	1. Enhancing precursor supply: Simultaneous knockdown of *PAH1* and *OPI1* improved ER function.<br>2. Knockdown both of *HRD1* and *OPI1* increased the storage capacity of lipid droplets.<br>3. The combined deletion of *UTH1* and *ECM33* enhanced the robustness of the engineered strain.	/	55.82 g/L<br>(5 L bioreactor)	[1]
S. cerevisiae	1. Employing computer-aided design: It provides a framework for prediction and mining of terpenoid exporters, which can be used to identify exporters of other terpenoids.<br>2. Extracellular secretion: the utilization of ATP-binding cassette transporter Pdr5 and oxysterol-binding homology protein Osh3 could promote squalene efflux. Overexpression of *Pdr5* and *Osh3* increased the secretion of squalene by 141.1-fold, and the total amount of squalene reached 3242.33 mg/L in a 3 L bioreactor.		3242.33 mg/L<br>(3 L bioreactor)	[2]

S. cerevisiae	1. Enhancing precursor supply:<br>1.1 Overexpression and multi-copy integration of *tHMG1* resulted in the highest yield of squalene of 550.89 mg/L.<br>1.2 By overexpressing *TGL3*, *TGL4*, *FAA*$_1$, and *FAA*$_4$, the supply of acetyl-CoA was improved and squalene synthesis was promoted.<br>2. Alleviating consumption pathways: N-degron labeling technology was used to reduce the activity of Erg1 protein by controlled degradation while maintaining normal cell growth.	1024.88 mg/L	6.53 g/L<br>(5 L bioreactor)	[3]
S. cerevisiae	1. Enhancing precursor supply:<br>1.1 Tagging MLS to the N-terminus of the gene introduced the entire MVA pathway into mitochondria and improved acetyl-CoA flux, then the key genes such as *ERG20* and *ERG9* were further overexpressed.<br>1.2 Organelle engineering: overexpression of key genes such as *ERG19* and *IDI1* reduced metabolic toxicity in mitochondria. Finally, cytoplasmic and mitochondrial engineering were combined to overexpress *tHMG1* to enhance the effect of the MVA pathway.	3000.63 ± 75.3 mg/L	21.1 g/L<br>(5 L bioreactor)	[4]
S. cerevisiae	1. Enhancing precursor supply:<br>1.1 Organelle engineering: Enhance the expression of *ERG10, ERG13, tHMG1, NADH-HMGR, ERG12, ERG8, MVD1, IDI1, ERG20, ERG9, ANT1* in the	1698.02 mg/L	11.00 g/L<br>(5 L bioreactor)	[5]

	peroxisome and dual cytoplasmic-peroxisomal engineering to overproduce squalene.<br>2. Cofactor engineering: Introduce heterologous efficient enzyme to relieve cofactor pressure by enhancing the expression of NADH-HMGR from *Silicabacter pomeroyi*. And overexpression of *IDP2* and *IDP3* provided sufficient NADPH to the squalene synthesis pathway in the middle compartment of the peroxisome.<br>3. Alleviating consumption pathways: The excessive consumption of squalene was reduced by replacing the *pERG1* with *pHXT1*.			
Y. lipolytica	1. Extracellular secretion:<br>1.1 A series of ABC transporters and oxosterol-binding proteins (OSH) were screened, among which the combination of SNQ2 and OSH3 showed the most significant secretion of squalene.<br>1.2 A carrier protein-mediated metabolite transport system was designed by fusing the binding domain of OSH3 with a secretory signal peptide.<br>2. Enhancing precursor supply: overexpression of *AtoB, HMGS, HMGR, ERG12, ERG8, ERG19, IDI, ERG20, ACL1, ACL2, YHM2, AMPD, EfMvaE, EfMvaS*^{A110G}, the yield of squalene was 160-fold higher than that of Po1f.	/	12.61 g/L (3.43 g/L extracellular secretion)<br>(3 L bioreactor)	[6]
Y. lipolytica	1. Enhancing precursor supply:	/	32.8 g/L<br>(3 L bioreactor)	[7]

	1.1 Overexpression of *ERG10, ERG13, tHMGR, ERG12, ERG8, ERG19, IDI, ERG20, ERG9* and introduced *MvaE* and *MvaS* genes to enhance metabolic flux in the upstream pathway.<br>1.2 Squalene compartmentalization in peroxisomes.			
Y. lipolytica	1. Enhancing precursor supply:<br>1.1. Key gene screening and multicopy integration in the MVA pathway: *tHMG1, IDI1*, and *ERG9*.<br>1.2 Organelle engineering: the entire MVA pathway has been integrated into the peroxisomal and cytoplasm-peroxisome engineering (peroxisomal signal peptide screening) for overexpression and co-expression of *POT1, PXA1/2, and POX1/2* to prevent excessive lipid synthesis from consuming carbon flux in the MVA pathway.	2549.1mg/L	51.2 g/L<br>(5 L bioreactor)	[8]
Y. lipolytica	1. The remodeling of homologous recombination efficiency results in a 10-fold increase in the rate of homologous recombination.<br>2. Enhancing precursor supply:<br>2.1 Organelle engineering: the entire MVA pathway has been integrated into the chromosome to enhance squalene production.<br>2.2 *ACL, MvaE* and *tHMG1* were overexpressed, and *ACS*^{L641P} was integrated to enhance acetyl-CoA utilization by the MVA pathway.	2.4 g/L	35 g/L<br>(5 L bioreactor)	[9]

	3. Terbinafine was added at a certain concentration.			
Y. lipolytica	1. Enhancing precursor supply:<br>1.1 The efficiency of acetyl-CoA synthesis is improved by adjusting the carbon flux of pyruvate dehydrogenation to promote the binding of acetate to CoA or the cleavage of citrate.<br>1.2 Overexpression of *Salmonella enterica (acs*)* or *ylACL1* increased intracellular acetyl- CoA levels by more than 50%.<br>1.3 By iterative chromosome integration of *ylHMG1, acs** and *ylACL1*, squalene production was increased by 16.4-fold.	10 mg/g DCW	/	[10]

References:

[1] Tang S, Gao W, Guo Q, Wei D; Wang FQ. Orchestrating multiple subcellular organelles of *Saccharomyces cerevisiae* for efficient production of squalene. Bioresour Technol 2025;424:132294. https://doi.org/10.1016/j.biortech.2025.132294.

[2] Liu J, Wang X, Jin K, Liu Y, Li J, Du G, Lv X; Liu L. *In Silico* prediction and mining of exporters for secretory bioproduction of terpenoids in *Saccharomyces cerevisiae*. ACS Synth. Biol. 2023;12:863-876. https://doi.org/10.1021/acssynbio.2c00673.

[3] Son S-H, Kim J-E, Moon SY, Jang I-S, Yu BJ; Lee JY. Metabolic recycling of storage lipids promotes squalene biosynthesis in yeast. Biotechnol. Biofuels Bioprod. 2022;15:108. https://doi.org/10.1186/s13068-022-02208-9.

[4] Zhu Z-T, Du M-M, Gao B, Tao X-Y, Zhao M, Ren Y-H, Wang F-Q; Wei D-Z. Metabolic compartmentalization in yeast mitochondria: burden and solution for squalene overproduction. Metab. Eng. 2021;68:232-245. https://doi.org/10.1016/j.ymben.2021.10.011.

[5] Liu G-S, Li T, Zhou W, Jiang M, Tao X-Y, Liu M, Zhao M, Ren Y-H, Gao B, Wang F-Q; Wei D-Z. The yeast peroxisome: a dynamic storage depot and subcellular factory for squalene overproduction. Metab. Eng. 2020;57:151-161. https://doi.org/10.1016/j.ymben.2019.11.001.

[6] Chai L, Che J, Liu X, Wang Z, Qi Q; Hou J. Secretory and metabolic engineering of squalene in *Yarrowia lipolytica.* Bioresour. Technol. 2025;421:132171. https://doi.org 10.1016/j.biortech.2025.132171.

[7] Ma Y, Shang Y; Stephanopoulos G. Engineering peroxisomal biosynthetic pathways for maximization of triterpene production in *Yarrowia lipolytica*. Proceedings of the National Academy of Sciences 2024;121:e2314798121. https://doi.org/doi:10.1073/pnas.2314798121.

[8] Ning Y, Liu M, Ru Z, Zeng W, Liu S; Zhou J. Efficient synthesis of squalene by cytoplasmic-peroxisomal engineering and regulating lipid metabolism in *Yarrowia lipolytica*. Bioresour. Technol. 2024;395:130379. https://doi.org/10.1016/j.biortech.2024.130379.

[9] Xu M, Yang N, Pan J, Hua Q, Li C-X; Xu J-H. Remodeling the homologous recombination mechanism of *Yarrowia lipolytica* for high-level biosynthesis of squalene. J. Agric. Food. Chem. 2024;72:9984-9993. https://doi.org/10.1021/acs.jafc.4c01779.

[10] Huang Y-Y, Jian X-X, Lv Y-B, Nian K-Q, Gao Q, Chen J, Wei L-J; Hua Q. Enhanced squalene biosynthesis in *Yarrowia lipolytica* based on metabolically engineered acetyl-CoA metabolism. J. Biotechnol. 2018;281:106-114. https://doi.org/10.1016/j.jbiotec.2018.07.001.
